# Administration of Human Chorionic Gonadotropin Combined with Phenylbutazone at the Time of Embryo Transfer Synergistically Improves Pregnancy Rates in Dromedary Camels

**DOI:** 10.3390/vetsci13010085

**Published:** 2026-01-15

**Authors:** Mahmoud Moussa, Salahaddin Ahmed, Mohamed Elbaz, Kamaal Pasha

**Affiliations:** 1 Emirates Smart Camel Center, Umm Al Quwain P.O. Box 7600, United Arab Emirates; 2 Department of Theriogenology, Faculty of Veterinary Medicine, Suez Canal University, Ismailia 41522, Egypt

**Keywords:** dromedary camels, embryo transfer, hCG, phenylbutazone, eCG

## Abstract

Effective luteal support is crucial for successful pregnancy following embryo transfer in dromedary camels, highlighting the importance of maintaining adequate luteal function. This study compared three luteal support protocols for pregnancy rates after embryo transfer (ET) in dromedary camels. Recipients with a single ovarian follicle (14~16 mm) were assigned into: (1) untreated control, (2) Phenylbutazone (PBZ) at 3.7 mg/kg IV prior to ET, or (3) human chorionic gonadotropin (hCG) 2000 IU plus 3.7 mg/kg IV PBZ prior to ET. Recipients that showed ovarian inactivity were treated with an injection of 1800 IU of equine chorionic gonadotropin (eCG) after progesterone (P_4_) priming. Pregnancy was assessed on days 10 (P_4_ assay) and 60 (transrectal ultrasonography) post-ET. Pregnancy rates on day 10 were higher in the hCG + PBZ (73.3%) and eCG (68.0%) groups than in the PBZ (44.0%) and control (35.0%) groups (*p* < 0.05). By day 60, the hCG + PBZ group exhibited the highest pregnancy maintenance rate (81.8%) and the lowest embryonic loss rate (18.2%), compared with eCG (53.0%; 47.0%), PBZ (54.5%; 45.5%), and control (57.1%; 42.9%) groups, respectively (*p* < 0.05). However, there was no significant difference among the eCG, PBZ, and control groups in embryonic loss rates. In conclusion, hCG synergizes with PBZ to enhance corpus luteum function, uterine receptivity, and pregnancy maintenance more effectively than PBZ or eCG.

## Simple Summary

Pregnancy rates after embryo transfer in dromedary camels vary widely due to biological and management-related factors, with inadequate luteal function being a key limiting factor. This study provides practical evidence that optimizing luteal support at embryo transfer can improve pregnancy outcomes. Recipient camels were allocated to four groups: a control group receiving no luteal support; Phenylbutazone alone; a combination of human chorionic gonadotropin (hCG) and Phenylbutazone; and stimulation with equine chorionic gonadotropin (eCG). The combined administration of hCG and Phenylbutazone at embryo transfer yielded the highest pregnancy rates at day 60 post-transfer, compared with the control and other treatment groups. From a practical perspective, this protocol offers a feasible and cost-effective alternative to eCG-based stimulation, which requires repeated handling, multiple injections and higher costs and is known to have several drawbacks in camelid reproduction. The improved outcomes are likely due to the synergistic anti-luteolytic effect of Phenylbutazone and the luteotrophic action of hCG, resulting in enhanced and sustained luteal function. Additionally, hCG may improve endometrial receptivity, further supporting embryo survival. Overall, these findings offer a practical, field-applicable strategy to enhance the efficiency and success of camel embryo transfer programs, particularly under large-scale production conditions.

## 1. Introduction

Embryo transfer (ET) in dromedary camels was initially applied to enhance genetic improvement, reproductive efficiency, and the conservation of genetically superior camels. It has since become a widely used technique for commercial purposes. However, pregnancy rates in camels vary widely, from 0% to 67%, and are influenced by several factors, including the season, the quality and quantity of corpora lutea in recipients, the age and quality of the embryo, and synchrony between donor and recipient [1,2]. Pregnancy losses after ET in camels may be influenced by donor and recipient age [3], embryo quality [4], and the year of the study [5], with suboptimal luteal function being a major contributing factor [5]. Both sub- and supra-optimal progesterone (P_4_) concentrations have been reported to adversely affect embryonic survival in cattle [6]. Thus, luteal support and maintenance of an optimal uterine environment following ET are essential for embryonic development, uterine receptivity, and higher pregnancy rates in dromedary camels. Previous approaches involved daily administration of exogenous P_4_ during the peri-ET period [7]. However, P_4_ supplementation, either by daily injection in camels [2] or by an intravaginal progesterone-releasing device (CIDR) in cattle [8], has not improved pregnancy rates. Furthermore, in cattle, several studies have shown that early exogenous P_4_ administration during the estrus cycle adversely affects corpus luteum (CL) function, resulting in premature luteolysis and subsequent pregnancy loss [9,10]. Taken together, strategies that stimulate endogenous P_4_ production may be more effective than exogenous sources for improving pregnancy rates.

Another strategy for synchronizing ovulation between donors and recipients involves combining progesterone with equine chorionic gonadotropin (eCG) to stimulate multiple follicular growth and the subsequent formation of various corpora lutea (CLs), thereby enhancing endogenous P_4_ secretion and improving pregnancy rates [1]. However, such circumstances subject the uterus to supra-physiological levels of estradiol from multiple follicles and to high P_4_ produced by multiple CLs, which can adversely affect embryo development, embryo-maternal communication, and implantation in humans and mice [11,12,13,14,15].

Non-steroidal anti-inflammatory drugs (NSAIDs) are commonly used during ET to enhance pregnancy rates in cows [16]. They inhibit prostaglandin synthesis, particularly PGF2α, thereby alleviating post-transfer inflammatory responses, delaying luteolysis, and maintaining luteal function [16]. The use of NSAIDs, such as flunixin meglumine (FM) or meloxicam, in ET protocols has shown promising results in cattle [8,17,18,19] and mares [20]. However, Skidmore et al. [2] reported no improvement in pregnancy rates with FM in camels. Notably, meclofenamic acid may offer advantages over FM in ET protocols by modulating prostaglandin signaling without affecting embryonic mobility [21], which is important in camelid reproduction, where embryonic migration within the uterus is critical for successful implantation [22]. Phenylbutazone (PBZ) is a traditionally used NSAID in veterinary medicine for its analgesic, anti-inflammatory, and antipyretic properties. It differs from other NSAIDs in its longer duration of action and potent inhibition of prostaglandin F_2_α synthesis. Unlike COX-2-selective agents such as meloxicam, PBZ non-selectively inhibits both COX-1 and COX-2 [23,24]. Its combined anti-inflammatory and anti-luteolytic effects, mediated by inhibiting prostaglandin F_2_α synthesis, make it particularly effective in maintaining luteal function and supporting pregnancy in large animals under field conditions by reducing uterine inflammation after embryo transfer [20]. However, its effects on pregnancy rates in camel ET programs have not been well investigated. Given its distinct pharmacological properties, we hypothesized that PBZ may influence uterine receptivity and embryo survival differently than FM or meloxicam.

In human-assisted reproduction, luteal-phase supplementation with human chorionic gonadotropin (hCG) has yielded superior pregnancy [25], live birth [26], and conception [27] rates than exogenous P4 administration. Furthermore, administration of hCG on day 5 post-ovulation enhanced luteal function, increased P_4_ synthesis, and improved conception rates in buffaloes [28], cattle [29,30,31,32], sheep (day 4 post AI) [33], and llamas [34]. Similarly, in felids, hCG stimulates P_4_ production in cultured luteal cells [35], suggesting that hCG-mediated luteal support may enhance reproductive outcomes in other induced ovulators, such as camels. This beneficial effect is attributed to hCG’s dual role in stimulating the CL to produce P_4_ [36] and directly enhancing endometrial receptivity during implantation [37,38]. This highlights the need to investigate alternative strategies to optimize ET outcomes in camelids, potentially focusing on embryo-endometrial synchrony or novel luteotrophic support strategies. Despite its fundamental role in camel reproduction, the potential benefits of hCG and its interactions with NSAIDs in camel ET remain unexplored. Thus, this study aimed to compare the efficacy of three luteal support protocols on pregnancy rates in dromedary camel recipients following ET: (1) PBZ alone, (2) a combination of hCG and PBZ, and (3) eCG-stimulated recipients.

## 2. Materials and Methods

### 2.1. Animals, Housing, and Feeding

This study was conducted at the Emirates Smart Camel Center in Umm Al Quwain, United Arab Emirates, during the natural breeding season (October 2024 to March 2025). All procedures were performed in accordance with institutional animal welfare guidelines and relevant national regulations (approval number: 80/23). Twenty-five dromedary camel donors (Camelus dromedarius), aged 10–15 years, and 100 recipients, aged 8–12 years, were enrolled. All animals underwent thorough health screening and received prophylactic antiparasitic and trypanocide treatment 15–20 days before the start of the experiments. Subsequently, all females underwent breeding soundness evaluations, including transrectal ultrasonographic examination of the reproductive tract (MyLab Delta, Esaote, Genova, Italy) and vaginal inspection to assess genital health and function. Only clinically healthy camelids with normal reproductive status were enrolled. Animals with reproductive abnormalities, such as ovarian dysfunction, uterine pathology, or a history of reproductive disorders, were excluded. All selected animals were treated prophylactically with an intrauterine antibiotic (Metricure^®^, MSD Animal Health, Boxmeer, The Netherlands) seven days before ET to minimize the risk of uterine infection and optimize the uterine environment for embryo implantation. Female camels were housed by ownership in separate pens accommodating 6–8 animals each, with pen sizes ranging from 70 to 100 m^2^. Four fertile males (aged 8–15 years) with documented histories of successful natural mating were housed individually in pens measuring 8 × 9 m^2^. Shaded shelters and ventilation were provided to mitigate heat stress. All animals were maintained under standardized nutritional and management protocols. The daily diet consisted of Rhodes grass (4.0 kg/head), wheat bran (2.0 kg/head), concentrate feed containing 14% crude protein (2.0 kg/head), and dried alfalfa (2.0 kg/head). Fresh water and mineral blocks were available ad libitum throughout the study.

### 2.2. Management of Donors

Donor camels with ovarian follicles measuring 14~16 mm in diameter were administered 20 µg of a GnRH analogue (Buserelina Zoovet^®^, Laboratorio Zoovet, Santa Fe, Argentina) intramuscularly to induce ovulation. On Day 4 post-ovulation, donors received a single intramuscular injection of 3000 IU eCG [39] (Novormon^®^, Syntex S.A., Santa Fe, Argentina), designated as Day 1. This was followed by intramuscular administration of 500 µg Cloprostenol (Bioestrovet^®^, Vétoquinol, Lure, France) on Day 4 after the eCG injection. Donors were then screened and mated when the majority of ovarian follicles had reached 14~16 mm. To enhance fertilization success, donor camels were mated twice at 12 h intervals. Immediately after the first mating, each donor received an intramuscular injection of 40 µg of a GnRH analogue. The first day of mating was designated as Day 0. Ovarian response was monitored by transrectal ultrasonography on the day of ET to confirm ovulation and assess ovarian activity; donors who did not ovulate were excluded from the study. Consequently, only 25 donor camels with confirmed ovulation were included.

### 2.3. Management of Recipients

Recipient camels were examined by transrectal ultrasonography to assess ovarian status and uterine health. Only non-stimulated camels with a single ovarian follicle measuring 14~16 mm in diameter were assigned to the control, hCG + PBZ, and PBZ treatment groups. Recipients that showed no signs of ovarian activity were pretreated with a daily intramuscular injection of 100 mg of P_4_ (Proluten^®^, Livisto Animal Health, Zaragoza, El Salvador) for 10 days, followed by an injection of 1800 IU of eCG to stimulate follicular development [1]. All recipients were administered 20 µg of Buserelin acetate 24 h after the donors were mated to optimize pregnancy rates [2]. On the day of ET (Day 8 after the donors’ mating), recipients were re-evaluated to confirm ovulation and the presence of CLs.

### 2.4. Embryo Collection and Transfer

Embryos were recovered using non-surgical, transcervical uterine lavage. Animals were restrained, standing in a suitably designed crate, sedated via intravenous administration of xylazine (Xyla 2%^®^, Interchemie, Venray, The Netherlands), and the tail was secured with a bandage. The vulvar and perineal areas were cleansed with sterile cotton and disinfected with 70% ethanol. An 18–22-gauge Foley catheter (Minitube, Taipei, Taiwan) was inserted into the base of the uterine horn, and the balloon cuff was inflated with 30 mL of air to secure placement. The uterine horn was flushed repeatedly with 100 mL aliquots of a commercial flushing medium (CamelFlush^®^, Minitube, Smythesdale, Australia), for a total volume of 1000–1500 mL per donor. The effluent was then recovered by gravity flow into a sterile embryo filter (Minitube, Verona, WI, USA). Embryos were recovered from the filter and examined under a stereomicroscope at 20× magnification within a sterile laminar flow cabinet (IVFtech, Stenløse, Denmark). The average number of recovered embryos in this study was 6.8 ± 2.0, and a total of 170 embryos were recovered from 25 donors, of which 120 (70.58%) were classified as transferable. Hatched blastocysts were transferred to a 5-well dish containing holding medium (Bovihold^®^, Minitube, Smythesdale, Australia) and washed three times. Only embryos that were spherical, morphologically clear, and of medium size (601–985 µm) were selected for this study to ensure the best quality of transferred embryos and higher pregnancy rates [40]. Each selected embryo was aspirated individually into a 0.25 mL plastic straw and loaded into a sheathed embryo transfer gun. Recipients were sedated, and the perineum was cleaned and disinfected with 70% ethanol. Embryo transfer was performed using a standard transfer gun, which was guided through the cervix and directed into the left uterine horn, regardless of the ovulation side, and the embryo was deposited.

### 2.5. Experimental Design

**Figure 1:**
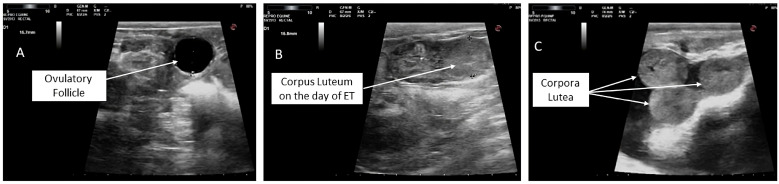
Ultrasound ovarian monitoring of the recipients: a single ovulatory follicle (15.7 mm) with no follicles on the other ovary (**A**); a corpus luteum (**B**) in unstimulated recipients (16.8 mm); and corpora lutea after eCG administration in stimulated recipients (**C**) on the day of embryo transfer.

**Figure 2:**
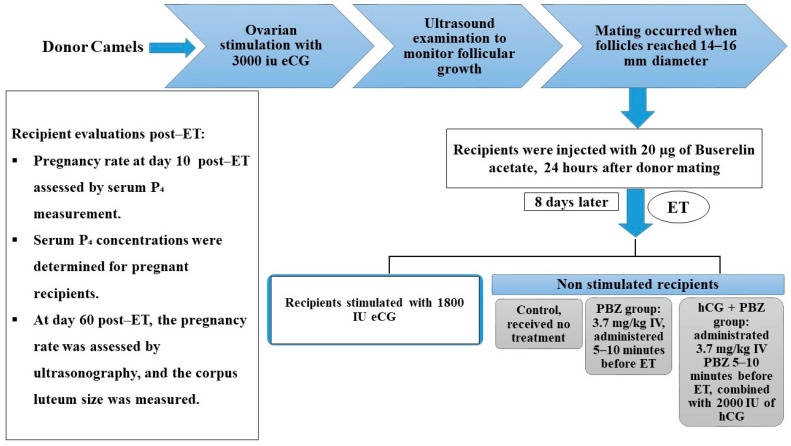
Schematic diagram summarizing the experimental model. ET, embryo transfer; P_4_, progesterone; hCG, human chorionic gonadotropin; PBZ, phenylbutazone; eCG, equine chorionic gonadotropin.

Recipient animals treated with eCG and confirmed to have developed 3 to 4 CLs at the time of ET were selected, whereas recipients with a single active CL (Figure 1) and P_4_ levels ranging from 2.0 to 2.5 ng/mL at the time of ET were randomly assigned to one of three experimental groups (Figure 2):

1. Control group: received no treatment.
2. PBZ group: treated with a maintenance dose of 3.7 mg/kg IV, administered 5–10 min before ET, of a nonsteroidal anti-inflammatory drug (Butasyl^®^, Zoetis, Madrid, Spain). Because specific dosing guidelines for camels are limited, we followed the drug’s directions and used the recommended maintenance dose to ensure safety and efficacy.
3. hCG + PBZ group: received 3.7 mg/kg IV PBZ 5–10 min before ET, along with 2000 IU of hCG (Ovusyn^®^, Syntex S.A., Argentina) at the time of ET. It has been reported that doses from 1500 to 3300 IU can improve pregnancy rates in cattle when administered during the peri-transfer period [32]. Based on these findings and due to the lack of research on hCG dosing in camels, we chose a 2000 IU dose of hCG for this study.

Following ET, recipient animals were housed separately and were provided a nutritionally balanced diet comprising 2.0 kg wheat bran, 6.0 kg Rhodes grass, and 2.0 kg of a 10% protein concentrate per head daily, designed to meet their maintenance and gestational nutritional requirements. All animals had ad libitum access to clean water and mineral blocks throughout the study period. Pregnancy was assessed on day 10 post-ET by measuring serum P_4_ levels using an electrochemiluminescence immunoassay (Roche Cobas E411 immunoassay analyzer, a fully automated machine). Confirmation of a viable conceptus and measurement of corpus luteum size were performed by transrectal ultrasonography on day 60 post-transfer. At day 10 post-transfer, values around 3 ng/mL were considered pregnant, and values below 1 ng/mL were considered non-pregnant. [41]. This experiment was conducted in five replicates (5 donors per replicate).

### 2.6. Statistical Analysis

The results were analyzed using SPSS11.0 software (SPSS, Chicago, IL, USA). The differences between means were analyzed by one-way ANOVA. Differences in pregnancy rates between groups were compared using Fisher’s exact test. While differences in P4 levels were detected using Tukey post hoc testing. A probability level of *p* < 0.05 was considered to be statistically significant.

## 3. Results

### 3.1. The Effect of Different Luteal Support Protocols on the Pregnancy Rate at Day 10 Post ET

The impact of various luteal support protocols on recipient pregnancy rates was assessed on day 10 post-ET using serum P_4_ analysis. Our results showed that the hCG + PBZ group had the highest pregnancy rate (73.3%), while the eCG group had a pregnancy rate of 68.0%. Both rates were significantly higher than those in the PBZ (44.0%) and control (35.0%) groups (*p* < 0.05). However, there was no significant difference between the hCG + PBZ and eCG groups, nor between the PBZ and control groups (Table 1).

### 3.2. Serum Progesterone Level at Day 10 Post ET After Different Luteal Support Protocols

Serum P_4_ concentrations in pregnant recipients varied significantly across treatment groups. The eCG group had the highest mean P_4_ level (8.4 ± 0.5 ng/mL), which was significantly higher than in the control (2.9 ± 0.8 ng/mL), PBZ (3.0 ± 0.2 ng/mL), and hCG + PBZ (5.5 ± 0.6 ng/mL) groups (*p* < 0.05). Furthermore, the hCG + PBZ group had a significantly greater P_4_ level (*p* < 0.05) than the control and PBZ groups. Although these high P_4_ values after hCG treatment were anticipated, the range was more variable than in the control and PBZ groups. There was no significant difference between the control and PBZ groups (Table 2). These results indicate that administration of hCG at the time of ET significantly elevates circulating P4 concentrations. Moreover, the elevated P_4_ levels in the eCG group might explain the higher early pregnancy rate compared to the control or PBZ groups.

### 3.3. Pregnancy and Early Pregnancy Loss Rates, and Corpus Luteum Size at Day 60 Post-ET in Recipients After Various Luteal Support Strategies

**Figure 3:**
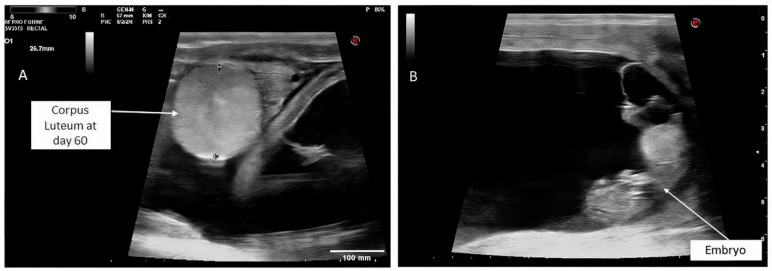
Ultrasound ovarian monitoring and pregnancy diagnosis at day 60 post ET in recipient camels: corpus luteum (26.7 mm) (**A**), and embryo (**B**).

By day 60 post-ET, the hCG + PBZ group had a significantly higher pregnancy rate (81.8%) than the control (57.1%), PBZ (54.5%), and eCG (53.0%) groups (*p* < 0.05) (Table 3). However, there was no significant difference among the eCG, PBZ, and control groups. Although the eCG treatment showed high initial pregnancy rates on day 10 post-ET, it was less effective at maintaining pregnancy than the hCG + PBZ group. Moreover, the pregnancy loss rate in the hCG + PBZ group was the lowest among the groups, at 18.2%, compared with the control (42.9%), PBZ (45.5%), and eCG (47.0%) groups (*p* < 0.05). However, there was no significant difference among the eCG, PBZ, and control groups in pregnancy loss rate. At day 60 post-embryo transfer, corpus luteum size did not differ significantly among experimental groups (*p* > 0.05). Mean values were 25 ± 0.6 mm in the control group, 24 ± 0.8 mm in the PBZ group, 26 ± 0.4 mm in the hCG + PBZ group, and 24 ± 0.1 mm in the eCG group (Table 3; Figure 3). These findings suggest that while eCG supports early pregnancy, hCG + PBZ is more effective at maintaining pregnancy viability and reducing early pregnancy losses. The data confirm that the improved pregnancy rate may be associated with enhanced luteal function and uterine receptivity.

## 4. Discussion

The present study demonstrated that the combined administration of hCG with PBZ significantly enhances the success of ET in the dromedary camel. The improved pregnancy rate could be attributed to enhanced uterine receptivity and luteal support provided by hCG along with PBZ, which counteract uterine inflammatory responses that interfere with embryo implantation. These findings are consistent with numerous studies reporting the essential role of hCG in stimulating P_4_ production by the CL [28,29,30,31,32,42,43]. Because camelids rely entirely on P_4_ from the CL to maintain pregnancy, it is essential to support luteal function and maintain adequate P_4_ secretion throughout gestation. Insufficient P_4_ concentrations in the early postovulatory period are associated with several adverse reproductive outcomes, including a suboptimal uterine environment and impaired conceptus development in cattle [44] and sheep [45], resulting in early embryonic loss. In buffalo, administration of hCG on day 5 post-ovulation induced hypertrophy of luteal cells, resulting in a high number of large luteal cells [46]. These cells produce 80% of luteal P_4_ [47] and, consequently, improve conception rates [28]. Similarly, in cattle, improved conception rates were observed following hCG treatment in the early luteal phase [29] and at the time of ET [42]. Despite this, our results showed that hCG had no effect on CL diameter, consistent with Kohne et al. [48], who treated mares early in the luteal phase and observed no change in CL size. In contrast, studies in cattle indicate that hCG can directly affect the corpus luteum, increasing its size and enhancing progesterone production [49]. The high embryonic survival rate in the hCG + PBZ group may also be attributed to the hormone’s direct effect on endometrial receptivity. Stimulation of LH receptors by hCG, which are present in uterine tissue, has been linked to enhanced endometrial secretory activity during the implantation window [37,38]. Taken together, the data indicate that the improved embryo survival rate after hCG administration may be due to both the luteotrophic effect, which results in optimal and sustained P_4_ production, and enhanced uterine receptivity.

Although P_4_ combined with eCG administration remains widely used for recipient preparation and synchronization [1], it has several drawbacks in camelid reproduction. Daily handling and injections make this approach labor-intensive and expensive, particularly at a large scale [50]. Additionally, the long half-life tends to induce ovarian overstimulation, asymmetric follicular growth, and the risk of anovulatory follicles; moreover, its antigenic properties further render the ovaries refractory, significantly diminishing ovarian responsiveness when repeatedly applied in subsequent cycles [5]. In the present study, while the eCG group exhibited a higher pregnancy rate than the PBZ and control groups at day 10 post-ET, the ability to maintain pregnancy was significantly reduced by day 60. This may be due to excessive follicular development, resulting in high estrogen levels that can lead to premature uterine gland activation, which ultimately compromises uterine receptivity and the embryo implantation process [51,52]. In addition, exposure of the uterus to supraphysiological levels of P_4_ produced by multiple corpora lutea may affect uterine receptivity and embryo survival [53]. The use of eCG can also result in asynchronous follicular growth and variable intervals to ovulation, increasing the risk of embryo-endometrial asynchrony [5], a major cause of ET failure. Although progesterone is a key regulator of uterine function and embryo development, the characteristics of the uterus during early embryonic loss and how systemic progesterone levels influence this period remain poorly understood [44]. The expression of uterine genes encoding the transport proteins, such as retinol-binding protein (RBP) and folate-binding protein (FBP), appears highly sensitive to systemic progesterone levels [44]. Elevated P_4_ levels, resulting from corpora lutea in the eCG group, may therefore impair uterine receptivity and disrupt embryo–maternal communication. Taken together, these findings indicate that eCG use adversely affects endometrial preparation and/or receptivity by altering steroid hormone levels relative to a natural cycle [13]. For this reason, despite being time-consuming [2], selection of cyclic recipients with mature ovarian follicles is recommended to ensure optimal P4 production and an adequate uterine environment, both of which are critical for successful embryo implantation and subsequent pregnancy maintenance.

Moreover, the results of incorporating NSAIDs into the ET process are inconsistent. We found that administering PBZ at the time of ET in camels did not improve the pregnancy rate. These findings are consistent with previous results using other NSAIDs, such as FM in camels [40] and meloxicam in alpaca [54]. In contrast, others have reported a positive impact in cattle [8,16,17,18] and mares [20]. However, these discrepancies between studies could be attributed to species-specific differences in luteal function or variations in the dosage and timing of NSAIDs administration. The observed results in our study might be attributed to PBZ’s long half-life, which may exert an excessive inhibitory effect on uterine prostaglandins, reducing uterine motility, thereby impairing embryo movement and maternal recognition of pregnancy [22,55], resulting in early embryonic loss. This suggests that while NSAIDs can maintain uterine function during ET by preventing subclinical endometritis [20], excessive inhibition could compromise early reproductive processes in camelid ET. Collectively, these findings imply that anti-inflammatory therapy alone may be insufficient without the support of a luteal function. It is likely that PBZ reduces PGF2α-mediated luteolysis; however, hCG synergistically maintains a functional CL. This combined treatment may offer a more effective strategy for improving ET success in camelid reproduction programs.

## 5. Conclusions

Our results suggest that administering hCG in combination with PBZ at the time of ET enhances the pregnancy rate in camels. The anti-luteolytic effect of PBZ, combined with luteotrophic support from hCG, provides more favorable, sustained luteal function, necessary for the successful establishment of pregnancy following ET in camels. Moreover, administration of hCG can also enhance endometrial receptivity. Future research should address the molecular mechanisms by which hCG affects uterine receptivity and whether further optimization of this protocol can be achieved with different hCG and PBZ doses.

## Figures and Tables

**Table 1:** The effect of different treatments (PBZ, hCG + PBZ, and eCG) compared to a control group on pregnancy rates at day 10 post ET in camels.

Groups	Number of Recipients (n)	Pregnant (n)	Pregnancy Rate (%)
Control	20	7	35.0% ^a^
PBZ	25	11	44.0% ^a^
hCG + PBZ	30	22	73.3% ^b^
eCG	25	17	68.0% ^b^

Groups with different letters (a or b) are significantly different (*p* < 0.05). Pregnancy rates at day 10 after embryo transfer were calculated by dividing the number of recipients diagnosed pregnant at day 10 (serum progesterone ≥ 3 ng/mL) by the total number of recipients who received embryos, then multiplying by 100.

**Table 2:** Progesterone assay in pregnant recipients treated with PBZ, hCG + PBZ, or eCG compared to a control group at day 10 following embryo transfer in camels.

Group	Pregnant Recipients (n)	Progesterone (ng/mL, Mean ± SD)
Control	7	2.9 ± 0.8 ^a^
PBZ	11	3.0 ± 0.2 ^a^
hCG + PBZ	22	5.5 ± 0.6 ^b^
eCG	17	8.4 ± 0.5 ^c^

Groups with different letters (a, b, or c) are significantly different (*p* < 0.05).

**Table 3:** Pregnancy and early pregnancy loss rates, and corpus luteum size on day 60 in recipients treated with PBZ, hCG + PBZ, or eCG, compared with a control group, following embryo transfer in camels.

Group	Pregnant Recipients at Day 10 Post ET (n)	Pregnant Recipients at Day 60 Post ET (n)	Pregnancy Rate at Day 60 Post ET (%)	Early Pregnancy Loss Rate (%)	Size of Corpus Luteum at Day 60 Post ET (mm)
Control	7	4	57.1 ^a^	42.8 ^a^	25 ± 0.6 ^a^
PBZ	11	6	54.5 ^a^	45.5 ^a^	24 ± 0.8 ^a^
hCG + PBZ	22	18	81.8 ^b^	18.1 ^b^	26 ± 0.4 ^a^
eCG	17	9	53.0 ^a^	47.0 ^a^	24 ± 0.1 ^a^

Groups with different letters (a or b) are significantly different (*p* < 0.05). Pregnancy rates at day 60 were calculated as (number of diagnosed pregnant recipients at day 60 by ultrasound/total number of pregnant recipients at day 10), then multiplying by 100. While early pregnancy loss rates were calculated as (number of pregnancies lost between days 10 and 60/total pregnant recipients at day 10), then multiplying by 100.

## Data Availability

The original contributions presented in this study are included in the article. Further inquiries can be directed to the corresponding author.

## Abbreviations

The following abbreviations are used in this manuscript:

ET	Embryo Transfer
PBZ	Phenylbutazone
hCG	Human chorionic gonadotropin
eCG	Equine chorionic gonadotropin
P_4_	Progesterone
CIDR	Intravaginal progesterone-releasing device
CL	Corpus luteum
NSAIDs	Non-steroidal anti-inflammatory drugs
FM	Flunixin meglumine
